# Implementing decision aids for cardiovascular disease prevention: stakeholder interviews and case studies in Australian primary care

**DOI:** 10.1186/s12875-023-02258-4

**Published:** 2024-02-03

**Authors:** Carissa Bonner, Samuel Cornell, Kristen Pickles, Carys Batcup, Carl de Wet, Mark Morgan, Kim Greaves, Denise O’Connor, Anna L Hawkes, Paul Crosland, Niamh Chapman, Jenny Doust

**Affiliations:** 1 Faculty of Medicine and Health, University of Sydney, Edward Ford Building, A27, Sydney, NSW 2006 Australia; 2 Gold Coast Hospital and Health Service, Gold Coast, QLD Australia; 3 Faculty of Health Sciences & Medicine, Bond University, Robina, QLD Australia; 4 Department of Cardiology, Sunshine Coast University Hospital, University of the Sunshine Coast, Birtinya, QLD Australia; 5 Department of Epidemiology and Preventive Medicine, School of Public Health and Preventive Medicine, Monash University, Melbourne, VIC Australia; 6 Monash-Cabrini Department of Musculoskeletal Health and Clinical Epidemiology, Cabrini Health, Malvern, VIC Australia; 7 School of Public Health, Faculty of Medicine, University of Queensland, Herston, QLD Australia; 8 Youth Mental Health and Technology, Brain and Mind Centre, Faculty of Medicine and Health, Translational Research Collective, University of Sydney, Sydney, NSW Australia; 9 Australian Women and Girls’ Health Research (AWaGHR) Centre, School of Public Health, Faculty of Medicine, University of Queensland, Herston, QLD Australia

**Keywords:** Cardiovascular disease, Prevention, Decision support, Shared decision making, Implementation, Primary care

## Abstract

**Background:**

Australian cardiovascular disease (CVD) prevention guidelines recommend absolute CVD risk assessment, but less than half of eligible patients have the required risk factors recorded due to fragmented implementation over the last decade. Co-designed decision aids for general practitioners (GPs) and consumers have been developed that improve knowledge barriers to guideline-recommended CVD risk assessment and management. This study used a stakeholder consultation process to identify and pilot test the feasibility of implementation strategies for these decision aids in Australian primary care.

**Methods:**

This mixed methods study included: (1) stakeholder consultation to map existing implementation strategies (2018-20); (2) interviews with 29 Primary Health Network (PHN) staff from all Australian states and territories to identify new implementation opportunities (2021); (3) pilot testing the feasibility of low, medium, and high resource implementation strategies (2019-21). Framework Analysis was used for qualitative data and Google analytics provided decision support usage data over time.

**Results:**

Informal stakeholder discussions indicated a need to partner with existing programs delivered by the Heart Foundation and PHNs. PHN interviews identified the importance of linking decision aids with GP education resources, quality improvement activities, and consumer-focused prevention programs. Participants highlighted the importance of integration with general practice processes, such as business models, workflows, medical records and clinical audit software. Specific implementation strategies were identified as feasible to pilot during COVID-19: (1) low resource: adding website links to local health area guidelines for clinicians and a Heart Foundation toolkit for primary care providers; (2) medium resource: presenting at GP education conferences and integrating the resources into audit and feedback reports; (3) high resource: auto-populate the risk assessment and decision aids from patient records via clinical audit software.

**Conclusions:**

This research identified a wide range of feasible strategies to implement decision aids for CVD risk assessment and management. The findings will inform the translation of new CVD guidelines in primary care. Future research will use economic evaluation to explore the added value of higher versus lower resource implementation strategies.

**Supplementary Information:**

The online version contains supplementary material available at 10.1186/s12875-023-02258-4.

## Background

Cardiovascular disease (CVD) prevention guidelines have evolved from a focus on individual risk factors, such as high blood pressure, to an absolute risk approach based on multiple risk factors to predict the chance of a cardiovascular event in the next 5–10 years [1–3]. Taking multiple risk factors into account, including non-modifiable risk factors such as age, is a more accurate way to predict an individual’s chance of having a heart attack or stroke [1]. Those at higher risk are more likely to benefit from taking blood pressure and cholesterol medication [4]. At the time of this study, Australian CVD risk assessment guidelines were based on the five year US Framingham risk equation [5]. This changed to the New Zealand PREDICT model in July 2023 [6], which includes additional risk factors such as current medication and location as an indication of socio-economic status. In the UK and US, CVD prevention guidelines are based on different ten year algorithms, including nationally relevant measures of socio-economic status (QRISK) and race (AHA) [5, 7].

In response to international CVD risk guideline changes, there has been a call to better integrate shared decision making into CVD prevention, as the relative benefit versus harm of preventive medication in this context depends on patient values [4, 8, 9] (e.g. attitudes towards preventive medication) and preferences (e.g. to try lifestyle change). Some patients will benefit from taking medication to lower blood pressure and cholesterol, but many won’t, so this needs to be balanced against costs and side effects. There is substantial evidence for the effectiveness of decision aids to support shared decision making across a wide range of health topics [10].

The gap between guideline algorithm changes and clinical practice is evident through misclassification of patients: undertreatment of high-risk patients and, conversely, overtreatment of low-risk patients [11, 12]. In Australia, more than 50% of eligible patients are missing the necessary risk factor data to conduct an absolute risk assessment [13]. Australia’s performance on CVD risk assessment compares poorly to nearby New Zealand (NZ), where absolute risk assessment has been reported to reach 90% of eligible New Zealanders [14]. This may be due to differences in the implementation strategies used to support guideline implementation.

In New Zealand, the guidelines were supported by consistent financial incentives and decision support integrated with general practice systems, including visual risk communication tools [14]. Implementation approaches to support uptake of CVD guidelines in Australian primary care include electronic decision support tools [15, 16] and quality improvement programs [17]. However, the effectiveness of these strategies was hampered by a failure to adapt decision support to guideline updates between 2009 and 2012, meaning that some high risk patients could be misclassified and miss out on preventive medication that could have reduced their risk of heart attack and stroke [18]. There were also numerous workflow and business model barriers to implementing CVD risk assessment in general practice [19]. Previous research has documented GP misunderstanding about the role of different risk factors in absolute risk guidelines, a sustained focus on single risk factors rather than absolute risk to inform treatment decisions [20], and difficulty communicating absolute risk to patients with lower health literacy [21–23]. Despite over 100 trials showing the effectiveness of patient decision aids to address these communication issues [10], implementation remains a challenge internationally [24]. Integration with clinical guidelines and GP software to enable point of care access to the tools is one approach to address this.

To address knowledge and communication barriers to the Australian CVD guidelines, we co-designed a decision aid with Australian general practitioners (GPs) to produce a single tool to apply the CVD prevention guideline algorithm, and discuss the individualised results with patients using a shared decision making approach [25]. The decision aid: (1) assesses individual risk according to the guidelines; (2) presents the results using visual icon arrays and multiple formats to support best practice risk communication; (3) links the result to the relevant management guidelines; and (4) generates interactive visuals and printable summary tables to help GPs discuss lifestyle and medication options with their patients. This was shown to increase GPs’ ability to identify high risk cases in a pre-post study, with high acceptability and intention to use the decision aid [25]. A patient version of the tool was also developed to address the needs of people with lower health literacy, which improved knowledge of risk and lifestyle change in a randomised trial [26].

### Aim

The introduction of new national guidelines with updated algorithms presented an opportunity to better coordinate the implementation of decision aids for CVD risk assessment and management. This research aimed to identify and pilot test the feasibility of different implementation strategies to integrate a co-designed decision aid in Australian primary care, using an informal stakeholder consultation process, formal stakeholder interviews, and opportunistic case studies within stakeholder programs.

## Methods

### Study overview

Implementation strategies were identified and pilot tested in three stages (Table S1). Stage 1 involved mapping potential implementation opportunities through informal consultation with stakeholders, based on existing programs in primary care to develop low, medium and high resource implementation strategies. Initial implementation strategies were identified informally through discussion notes. In stage 2 the feasibility of the strategies identified in stage 1 was explored via interviews with Primary Health Networks (PHNs) across all states and territories in Australia. A Framework Analysis process was used to thematically analyse transcripts, including the identification of different implementation strategies. More general findings about CVD prevention from the interviews have been reported elsewhere, along with the interview schedule [19]. The study team included diverse expertise including qualitative methods, CVD prevention, cardiology, general practice, behavioural science and health economics. For stage 3, selected implementation strategies were pilot tested in a real-world setting to explore the feasibility of the strategies to integrate decision support tools into primary care settings. More detailed testing of the software integration strategy has been reported elsewhere [27]. Ethics approval was provided by the University of Sydney Human Ethics Committee (#2020/255 for interviews and #2019/1047 for linking decision support to general practice software).

### Context

The context was changing over the course of this study in several ways: (1) the Heart Foundation was actively promoting a new government funded item to support GPs to do CVD risk assessment or “Heart Health Checks” (Medicare Benefits Schedule item numbers 699 and 177) [28]; (2) a national quality improvement program was introduced, including incentives for practices to share CVD risk data with PHNs [13]; and (3) there were variable COVID-19 impacts on general practice [29]. Due to these context issues, the implementation strategies were piloted in an opportunistic series of case studies in interested PHNs, rather than through a structured pilot adaptive trial as originally planned.

### Intervention materials

**Fig. 1:**
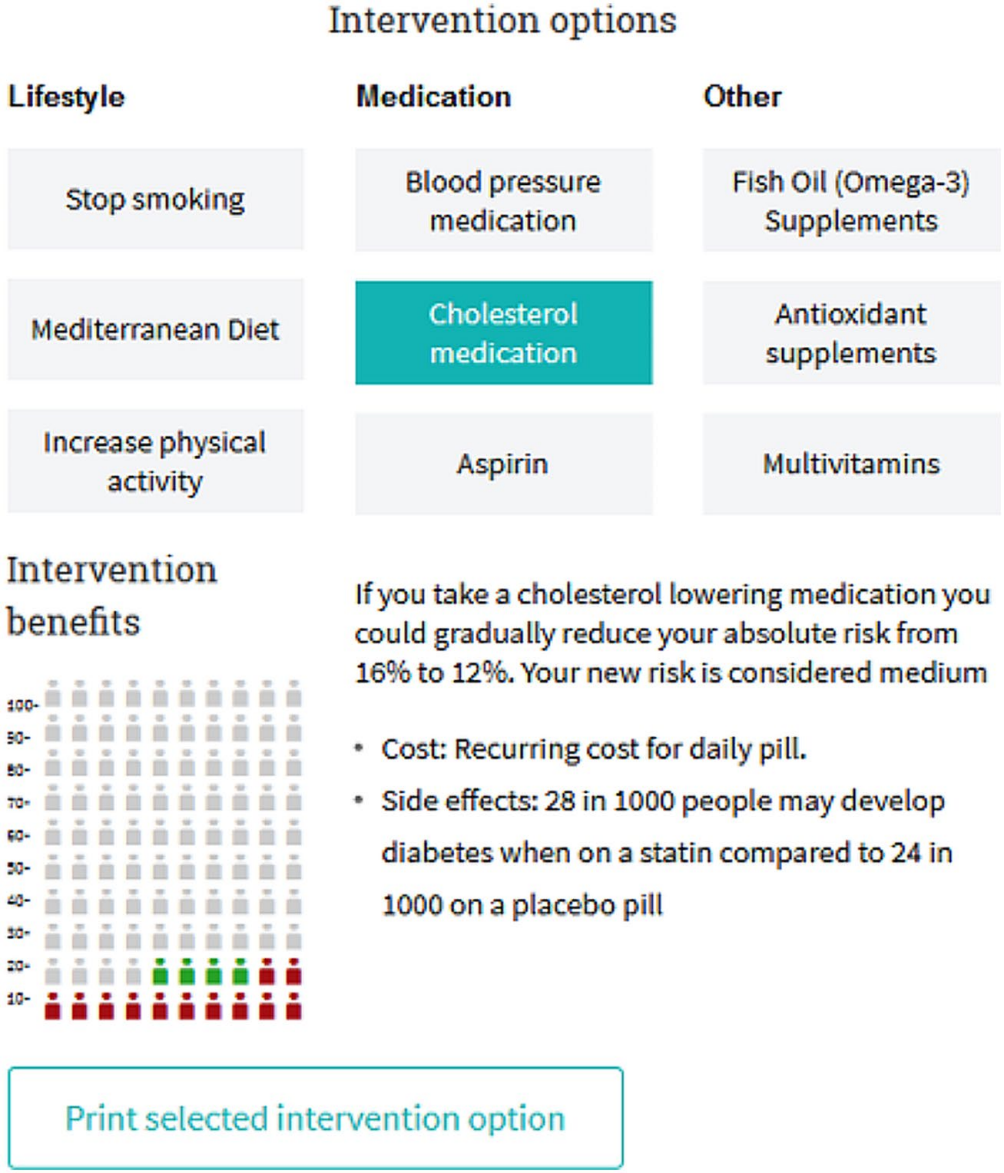
Decision aid for GPs to use during consultations

**Fig. 2:**
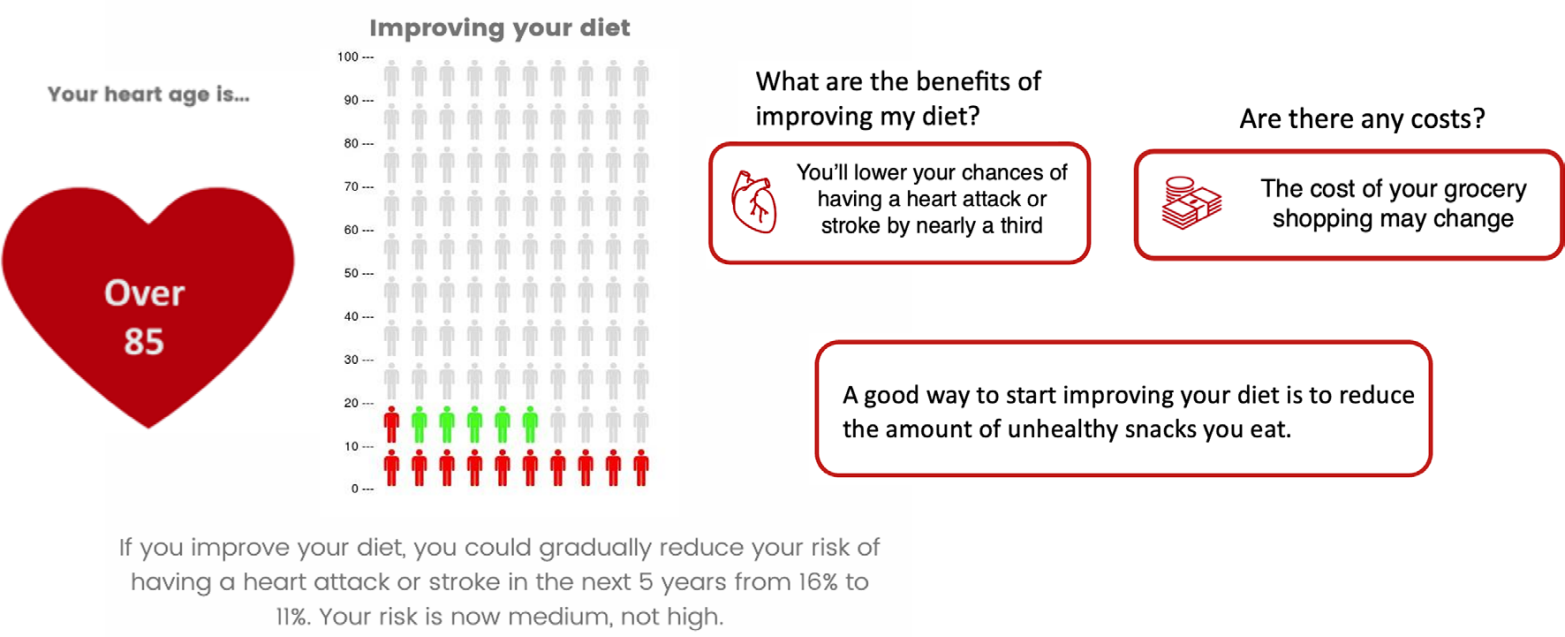
Decision aid for consumers to use before/after consultations

A co-designed decision support tool for GPs and patients was developed to support CVD risk assessment and management guidelines [25]. Specifically, we addressed the need to integrate assessment and management algorithms into a single tool for GPs, linked to best practice risk communication and decision aids for patients. We also developed a consumer version of the risk calculator and decision aid for people with varying health literacy levels, including evidence-based action plans to support lifestyle change [26]. Evaluations of these tools have been reported elsewhere [25, 26], and showed that they increase knowledge of guidelines-based risk categories for both GPs and consumers with varying health literacy needs. The GP version was tested with 98 GPs who used it over a one month period and reported increased pre to post knowledge of guidelines-based risk levels and high acceptability. The health literacy-sensitive consumer version of the tool was tested with 598 consumers over a one month period, with the health literacy-sensitive version shown to improve knowledge of risk and lifestyle change compared to current Heart Foundation materials in a randomised trial. See Figs. 1 and 2 for example content.

### Implementation strategies

Discussions with stakeholders in stages 1 and 2 identified a wide range of implementation strategies, requiring a range of resource levels. Low, medium and high resource strategies were selected by the advisory group based on feasibility given the context changes described above. Potential reach was explored using descriptive user time trends in Google analytics for the decision aid intervention website (www.auscvdrisk.com.au), to which all strategies were linked.

*Low resource strategies* included adding passive software links to existing websites with information about CVD prevention for clinicians. This involved demonstrating the co-designed decision support resources to Heart Foundation and PHN staff, who independently integrated this into their existing resources: PHN HealthPathways relating to CVD risk assessment and management, and the national toolkit for Heart Health Checks developed by the Heart Foundation. For HealthPathways we worked with one metropolitan PHN to write a brief description of the online decision support tool in assessment and management pathways for clinicians, which were being updated to incorporate Heart Health Checks. The PHN had already employed a GP writer to undertake this work, so this incurred no additional costs.

*Medium resource strategies* included participating in regional GP education events and providing tailored feedback to PHNs and practices from clinical audits, which required additional staff time to usual processes. The first strategy was developing a workshop for an annual GP education conference run by a PHN in Victoria. The workshop focused on CVD prevention guidelines and our associated decision support, and was scheduled for an in person conference in March 2020 but postponed to a virtual conference in May 2021 due to COVID-19 restrictions. The workshop was offered as part of a two day conference for which GPs received continuing professional development points. The second strategy was to work within activities for the national Practice Incentives Program Quality Improvement (QI) program that PHNs were already engaged in. We asked participants in Stage 2 to provide examples of audit and feedback reports sent to practices in their region, and integrated information about our decision aid into the highest scoring report.

*High resource strategies* included developing integrated software to auto-populate decision support tools using different software systems, which required substantial staff time and information technology costs, including development and additional licensing costs to usual processes. The app could auto-populate our decision support tools from medical records in two different clinical audit systems, requiring a single click from the GP. This is described in more detail in a separate paper [27].

## Results

### Stage 1. Informal stakeholder consultation

Table 1 summarises key stakeholders, potential implementation strategies, and feasibility issues for the Australian primary care context; documented through informal discussion notes after meetings. Stakeholders identified a key role for Primary Health Networks (PHNs) in implementing CVD risk assessment and management guidelines, as one of their key roles is to support general practices in their local region. This includes education, quality improvement initiatives and commissioned services that sometimes includes CVD prevention. Industry engagement to integrate the decision support tools with clinical management software was an additional implementation strategy identified to facilitate ease of access and audit and feedback for quality improvement. Finally, stakeholders identified ways to engage consumers directly including leveraging existing public health programs such as the Heart Foundation Heart Health Check campaigns.

**Table 1:** Strategies, cost and feasibility issues for each stakeholder group

Target group	Strategies	Cost issues	Feasibility issues
**Primary Health Networks**	- GP Liaison Roles - GP education - QI programs - Practice software	- PHN staff time - PIP QI payments - Ongoing licenses	- CVD prevention is not always a specific priority area (e.g. during COVID-19) - Software licenses not consistent
**General Practices**	- Online calculators - Linked calculators - MBS items - Recall methods - Toolkits	- Practice staff time for training - MBS reimbursement models - Costs of CPD review - Software licenses	- Business models don’t match funding models - Fragmented market for software solutions - New processes must fit with workflow
**Consumers**	- Online tools - Awareness campaigns - Community programs - Waiting room tools - Electronic health records	- Cost of media advertising - Short term funding for programs - Printing/equipment costs for waiting rooms	- How to develop a sustained approach - Competition for waiting room space - Privacy/access issues with EHR
**Health-related software developers**	- Medical record software - Clinical audit software - Recall software - Decision support software - Pathology report software	- Software development - Ongoing licenses - Printing/equipment costs for waiting rooms	- Fragmented markets for software requiring duplicate development - Competition for waiting room space

*Note*: CPD: continuing professional development; CVD: cardiovascular disease; EHR: electronic health record; GP: general practitioner; MBS: Medicare Benefits Schedule; PHN: Primary Health Network; PIP: Practice Incentives Program; QI: quality improvement

### Stage 2. PHN staff interviews

PHN staff (n = 29) interviews included a mix of frontline GP liaison staff and program management staff (Table 2). General findings on the role of prevention in PHN activities are reported elsewhere [19, 30]. The role of PHN staff to support the CVD guideline implementation strategies identified in stage 1 included leveraging the position of PHN staff within GP practice support programs; aligning the decision support tool to planned GP education and professional development programs; informing quality improvement programs to provide financial incentives to implement the decision support tool; and integrating the tool within existing PHN software (Table 3).

#### GP practice support

PHN staff highlighted the importance of considering the business model of the practice when implementing new initiatives. PHNs’ approaches to GP practice support were highly varied. Some PHNs focused on the most engaged practices who had indicated interest in specific programs or quality improvement issues. Others described a rolling six month engagement plan where staff would aim to contact many individual practices, typically this was based on current priorities of the PHN and available funding.

#### GP education for continuous professional development (CPD)

Professional development activities ranged from remote online modules that GPs could complete individually, to education conferences where GPs from the whole region would get together either in person or remotely. A major incentive to consider this approach as part of an implementation strategy was due to the CPD points that could be linked to the program and RACGP support.

#### Quality improvement (QI)

The main QI program involving CVD risk assessment was the federally funded Practice Incentives Program [13], including financial incentives to practices to share anonymized, aggregated, selected performance data each quarter and audit performance. This program includes a range of key health areas including CVD risk assessment, but practices may choose other areas to focus on for quality improvement. At the time of the study there was no requirement for practices to show a set level of improvement on those issues. QI goals were often vague with inconsistent timeframes. Some participants described starting with very small achievable goals and building up over time.

#### Practice software

There were two main software licences provided to practices via PHNs that were relevant to CVD prevention at the time of the study: HealthPathways provided information about CVD risk assessment and management with local referral pathways, and clinical audit software was used by practices to run their own reports, share data with PHNs, and access integrated decision support tools (e.g. auto-populated clinical algorithms, including for CVD risk assessment).

**Table 2:** Interview participant characteristics

Characteristic	Interviews n (%)	Number of (%) PHNs interviewed from each jurisdiction
State or Territory		
New South Wales	9 (31%)	4 (40%)
Western Australia	7 (24%)	3 (100%)
Queensland	4 (14%)	4 (57%)
Victoria	3 (10%)	3 (50%)
South Australia	2 (7%)	2 (100%)
Northern Territory	2 (7%)	1 (100%)
Tasmania	1 (3%)	1 (100%)
Australian Capital Territory	1 (3%)	1 (100%)
**Total**	29 (100%)	19 (61%)
**Area**	**Interviews n (%)**	
Regional	11 (38%)	
Metropolitan	8 (28%)	
Mixed (Western Australia, Northern Territory, Tasmania)^*^	10 (34%)	
**Total**	29 (100%)	
**Professional roles**	**Interviews n (%)**	
Managers of GP-related programs	21 (72%)	
Frontline staff working with GPs	8 (28%)	
**Total**	29 (100%)	

*Mixed regions included areas where PHNs cover both regional and metropolitan areas (e.g. Tasmania only has 1 PHN covering all cities and regions)

**Table 3:** Themes and illustrative quotes

Quotes to illustrate key implementation strategies at PHN level
Leverage GP practice support programs
*“We have to see all practices in a 6 month period - all of the practices… engagement is a continual process - once you are finished with a cycle you start again”. (interview 3, regional)*
*“When PHNs go to practices to introduce new programs – they need to be able to show to the practice the return on investment they would have, the health benefit and health outcome for patients.” (interview 19, regional)*
*“And the model I see working best is if, um, you can still keep a GP to a 15-minute appointment, happy days. But you could also tie that in with a 15-minute appointment with the nurse who could (commence?), have a lot of the dialogue, complete by the doctor” (interview 21, regional)”*
**Align with GP professional development**
*“We do quite a few sort of education events. So we either do clinical, er, in the past we did clinical updates, which could be whole day… um, events or evening. Um, and that’s pretty much all online now.” (interview 5, mixed region)*
*“I think education and creating more awareness to the GPs. In terms of these tools getting for them to, you know, have, it’s very difficult, for GPs. It’s not that easy…it goes through word of mouth as well. Of course. If anything is coming from our RACGP and then it actually helps the GPs to get on top of it. Not straightaway I would say. But sooner. Yeah.” (interview 3, regional)*
**Inform quality improvement programs**
*“because of PIPQI there is now a financial incentive for GPs to work in areas they wouldn’t have previously considered” (interview 2, metropolitan)*
*“They can self-report on their own CPD, but it would be like say a 12 week, a 12 week program or 6-month program where we would take them through PDSAs and get them to look at their data and improve their data, you know? Where that data is missing, you know? When your next person comes in the next time undertake those data checks, you know?” (interview 29, metropolitan)*
**Integrate within existing PHN software**
*“HealthPathways is always a good thing. I don’t think it’s necessarily a prevention thing but it’s an avenue for general practices to learn a little bit more about cardiovascular disease and how they can manage it and how they can sort of refer the patients onwards…CAT [clinical audit software] is a nice easy one, which all the PHNs are fairly familiar with and so we can kind of like go in and say, ok, let’s filter for these particular patients. Is there, a specific group of patients that you want to look at?” (interview 9, metropolitan)*

### Stage 3: pilot testing the feasibility of selected implementation strategies

Stage 3 tested a range of implementation strategies identified in stages 1 and 2. Table 4 summarises the implementation strategies that were pilot tested as case studies in stage 3, with change in Google analytics tracked via the intervention website.

#### Website links

Adding a link to the clinical resource webpage in one PHN resulted in an increase of 84 new website users in the month after implementation, which led to further website referrals from 16 unique HealthPathways in 4 Australian states (Vic, NSW, QLD, and Tas) and 1 from New Zealand over the course of the study. Referrals from the Heart Health Check toolkit led to 85 new monthly website users once the decision aid was integrated.

#### GP education activities

Fifty GPs attended the CVD prevention workshop that featured the decision support tool. For the QI strategy, eight PHN audit and feedback reports were assessed for quality against a best practice checklist (see Appendix). We integrated information about our decision support resources into the highest quality report, and this was sent by the PHN to 360 practices. As the two education strategies were conducted in the same month (May 2021) it’s not possible to separate their effect, but website visits increased from 349 in April to 469 in June.

#### Decision support software

This is reported in full detail elsewhere but a brief summary of implementation issues is provided here. Despite obtaining consent from nine practices initially, many withdrew when offered access to the decision support software due to COVID-19 related capacity issues. This left four practices that consented and accessed the intervention. Contrasting quotes to illustrate implementation drivers are below.

Example practice with high engagement: *“I think it’s great how it’s like, it just pulls all your data. A lot of things that you don’t have to really add in extra stuff, if need be. I would love if the chat tool actually grabbed all the other nutritional stuff, as well, and the family history…We’ve already inputted in, but it just doesn’t grab that. Yeah. So it would be great if it could do that as well. I just love anything that seamlessly crosses over. It’s just it’s amazing. I love digital health stuff.”*

Example practice with low engagement: “*unfortunately they [the doctors] have not really used the app. COVID issues took their focus and priority and there were issues of TopBar not keeping logged in, which meant they forgot to use it…. Sadly as for many other things, COVID has got in the way.”*

Highest individual use was in October when all four practices had access to the app, with 11 users (5 GPs, 4 nurses, 2 other), and highest overall use was in November with 285 sessions recorded in the app across four practices. There were 861 total sessions over a six month period from August 2021 to January 2022 but monthly use was highly variable by practice and over time.

**Table 4:** Case studies to pilot test the feasibility of implementation strategies

Implementation strategy	Date	Resource level	Summary of impact
Referral to website from HealthPathways	Oct 2019	Low – integrated with usual clinical updates in 1 PHN	Monthly new users increased from 111 in Sep to 195 in Nov. Further engagement with PHNs led to 578 total user referrals from 16 unique HealthPathways in 4 Australian states (Vic, NSW, QLD, and Tas) and one from New Zealand, from Sep 2019-Dec 2021.
Heart Foundation referrals from Heart Health Check Toolkit	Feb 2021	Low – integrated with existing program plans	Monthly new users increased from 106 in Jan to 191 in March. There were 152 total referrals from the Heart Foundation from Feb-Dec 2021.
GP education for CPD points	May 2021	Medium – CPD points application and presentation time	The workshop reached 50 GPs registered for an education conference for which they received CPD points, with a recorded video made available the next day.
Quality improvement reports	May 2021	Medium – requires data extraction from practices and creation of report	The CHAT-GP trial and website link was sent to 360 practices in PHN via the monthly PIP QI report.
App to integrate decision support in two clinical audit systems	July 2021	High – requires software development, licenses, information technology support	3 practices using PENCS software engaged with the app 832 times; 1 practice using POLAR software engaged with the app 33 times.

*Note*: CHAT-GP: Research program to improve the Communication of Heart disease risk Assessment using Translational strategies in General Practice; CPD: Continuing professional development; GP: General practitioner; NSW: New South Wales, Australian state; PHN: Primary Health Network; PIP: Practice Incentives Program; QI: Quality Improvement; QLD: Queensland, Australian state; Tas: Tasmania, Australian state; Vic: Victoria, Australian state

## Discussion

**Fig. 3:**
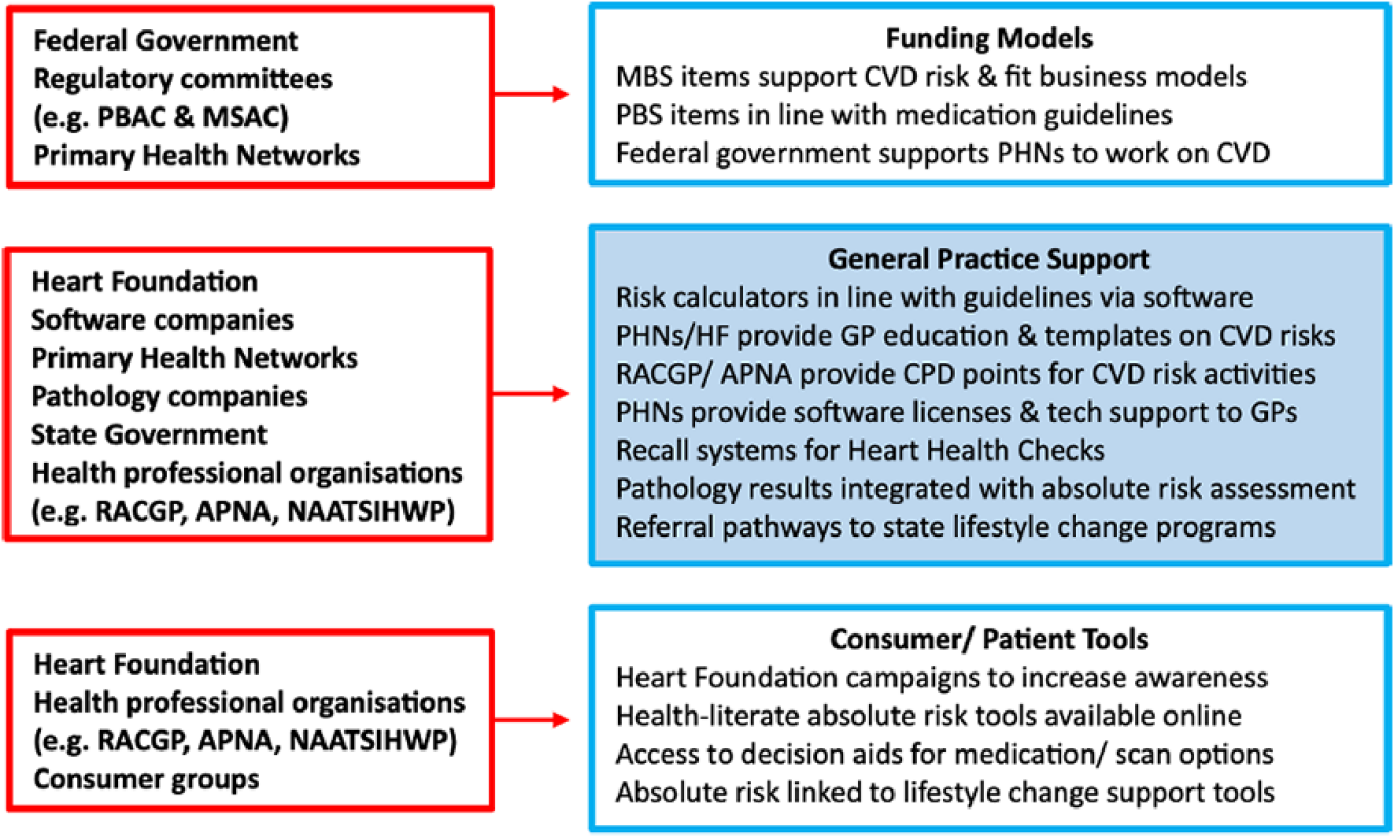
The role of different stakeholders in implementation strategies. *Note*: APNA: The Australian Primary Health Care Nurses Association; CPD: Continuing professional development; CVD: Cardiovascular diseases; GP: General practitioner; HF: Heart Foundation; MBS: Medicare Benefits Schedule; MSAC: Medical Services Advisory Committee; NAATSIHWP: The National Association of Aboriginal and Torres Strait Islander Health; PBAC: Pharmaceutical Benefits Advisory Committee; PBS: Pharmaceutical Benefits Scheme; PHN: Primary Health Network; RACGP: The Royal Australian College of General Practitioners Workers and Practitioners

This paper outlines a variety of ways that PHNs and other stakeholders can be involved in the implementation of decision support tools for CVD risk assessment and management guidelines. Many strategies have been shown to be feasible in the Australian primary care context, but the strategies have been isolated and sporadic since the 2012 management guidelines were released so the impact on CVD risk assessment data has been limited [13]. If a coordinated approach was used for the new guidelines we may achieve better implementation. This requires the involvement of PHNs, general practices, consumers and software industry partners to enable consistent messaging and tools. Figure 3 summarises relevant stakeholders for Australian primary care identified throughout the stakeholder consultation process.

This study provides new insights to inform the preparation and optimisation phases of the MOST approach [13] which is the framework for the broader program of research. The *preparation* phase identified feasible implementation strategies for CVD prevention decision support through stakeholder consultation and PHN staff interviews. This included regional GP practice support and education events, broader quality improvement and professional education programs, and software systems that integrate decision support tools with clinical workflows. The *optimisation* phase assessed the acceptability of implementation strategies to end users in previous work with GPs and consumers, and this paper provides a broader perspective showing that implementing decision support tools is feasible within existing resources at a PHN level. These findings will inform the *evaluation* phase in future research, aiming for an adaptive implementation trial to evaluate the value of adding targeted high/medium resource strategies (e.g. quality improvement reports sent to under-performing practices) to national low resource strategies (e.g. a standalone website allowing anyone to access decision support tools).

### Implications for a shared decision-making approach

The decision aids we developed are designed to support the clinical guidelines, but they are also based on a shared decision making approach [25, 26]. This is increasingly recognised as an important model for CVD prevention, where asymptomatic healthy patients need to understand their risk of heart attack or stroke in order to make an informed decision about lifestyle and/or medication approaches to reduce that risk [4, 8, 9, 31–33]. Incorporating the strategies identified in this study could support a shared decision making approach. From an evidence-based medicine perspective, we need to provide GPs and practice nurses with automated tools to quickly apply the latest evidence to their patients in line with Australian guidelines. These must be integrated with financial drivers, practice business models and clinical workflows in general practice, including the use of pathology reports [34]. This can be further supported by continued Federal Government funding for PHNs to work with practices on quality improvement and CVD prevention.

From a patient-centred communication skills perspective, GP education and better consumer tools are required to communicate the evidence to people with varying health literacy levels. These could be implemented via health literacy-sensitive risk calculators and decision aids, lifestyle change support tools such as action plans, and training for GPs and practice managers provided by professional organisations. The unique role of Aboriginal and Torres Strait Islander Health Workers and Practitioners should also be explored in the implementation of the revised CVD prevention guidelines [35]. To bring these two aspects together and facilitate shared decision making, we can use existing software to integrate both health professional and consumer tools into general practice. This would ideally involve working with software industry partners to integrate decision support tools with patient records, to enable quick and easy access to evidence-based advice.

### Future research

This study has focused on decision support as a key implementation strategy for CVD prevention guidelines. Other approaches to improve the use of CVD risk assessment in general practice have included asking patients to fill out pre-consultation risk assessment forms in waiting rooms [17, 36], using patient medical record and clinical audit software to auto-populate the risk assessment [37], and using self-directed blood pressure booths in pathology centres so the CVD risk assessment is provided in the pathology report to GPs [34]. All these approaches have been shown to be feasible in Australian primary care and support GPs to conduct CVD risk assessment, but they have been limited to trial settings and there has been no concerted effort to integrate multiple strategies into usual practice at a national level. If we can integrate the strategies already demonstrated to be feasible in Australian primary care, we can work towards better implementation of the revised CVD prevention guidelines [6]. The next goal of our research program is to: (1) adapt our decision support tools to the revised guidelines, and (2) conduct an adaptive trial to identify cost-effective approaches, as the next step in the MOST framework.

### Strengths and limitations

A strength of this study has been the wide stakeholder consultation, including investigators representing general practice and the Heart Foundation. We also used a theoretical framework that will enable us to identify cost-effective approaches in the next stage of our research program. A major limitation is that we cannot generalise these results to other health systems, as the Primary Health Network structure is very specific to the Australian context and this structure has changed several times in the past. In particular, the classification of lower versus higher resource implementation strategies in this study was based on integration with current programs that were already funded at the time of the study, which are likely to change over time and will be different in other primary care contexts. The feasibility outcomes for the implementation strategies were descriptive only, and we cannot disentangle the effect of strategies that were implemented in the same time period. The interviews demonstrated substantial impacts of COVID-19 on general practice that likely influenced our findings. Nevertheless, the paper provides methodological guidance and potential implementation approaches for algorithm-based guidelines in other countries that require decision support in primary care.

## Conclusion

Cardiovascular disease can be prevented by assessing and managing risk, and international prevention guidelines recommend using absolute risk calculators to guide treatment. Effective and evidence-based decision aids are essential for the successful implementation of CVD prevention guidelines in primary care, as they are based around complex algorithms that cannot be applied through clinical expertise or judgment alone. This program of research identified a range of potentially feasible strategies for implementing decision support for CVD risk assessment in Australian primary care systems. This will inform the development of a more coordinated national approach to translating new CVD prevention guidelines in primary care.

### Electronic supplementary material

Below is the link to the electronic supplementary material.

**Supplementary Material 1**: Checklist

**Supplementary Material 2**: Qualitative Checklist

**Supplementary Material 3**: Staff Semi-structured Interview Guide

**Supplementary Material 4**: Additional figure 1 : Quality of audit and feedback reports

**Supplementary Material 5**: Table S1: Implementation study stages

## Data Availability

The data are not available as not permitted under ethics approval for qualitative transcripts or practice data.

## Abbreviations

APNA: The Australian Primary Health Care Nurses Association
CESPHN: Central and Eastern Sydney Primary Health Network
CHAT-GP: Research program to improve the Communication of Heart disease risk Assessment using Translational strategies in General Practice
CPD: Continuing professional development
CVD: Cardiovascular disease
EHR: Electronic health record
GP: General practitioner
HF: Heart Foundation
MBS: Medicare Benefits Schedule
MOST: Multiphase Optimisation Strategy
MSAC: Medical Services Advisory Committee
NAATSIHWP: The National Association of Aboriginal and Torres Strait Islander Health Workers and Practitioners
NSW: New South Wales, Australian state
PBAC: Pharmaceutical Benefits Advisory Committee
PBS: Pharmaceutical Benefits Scheme
PHN: Primary Health Network; these are 31 region-specific health areas funded by the federal government to support primary care
PIP: Practice Incentives Program
PYLL: Potential Years of Life Lost
QI: Quality Improvement
QLD: Queensland, Australian state
RACGP: The Royal Australian College of General Practitioners
SDM: Shared decision making
Tas: Tasmania, Australian state
Vic: Victoria, Australian state
