# Supplementary material for: Implementing decision aids for cardiovascular disease prevention: stakeholder interviews and case studies in Australian primary care
Source: BMC Prim Care. 2024 Feb 3;25:49. doi: 10.1186/s12875-023-02258-4 (PMC10837956; doi:10.1186/s12875-023-02258-4)
Supplement: Supplementary file 1 — Supplementary Material 1: Checklist [file 12875_2023_2258_MOESM1_ESM.docx]

**Staff Semi-structured Interview Guide**

**Part 1. Context**

1. How would you describe your role within your PHN?
2. How do you engage with general practices?
3. What work does this predominantly encompass?
4. How does this role fit within the structure of the PHN?
5. What prevention activities are you aware of taking place in your PHN? (eg chronic disease management programme, chronic disease care plans, Healthy Habits app from RACGP, My Health for Life)
6. What is your perception/views of the risk assessment and prevention activities currently carried out in your PHN?
7. Are you aware of any similar programmes that are being conducted in other PHNs?
8. How would you describe knowledge transfer or engagement between your PHN and other PHNs regarding prevention activities?

**Part 2. Intervention readiness**

1. Do you have any thoughts on ways your PHN could increase engagement with general practices for CVD prevention activities? (HealthPathways, clinical audit tools, patient prevention summaries/reminder sheets, additional monitoring and reporting of performance measures, PIP QI).
2. How would you describe the receptiveness of General Practices in your PHN to engage in risk assessment or prevention programmes?
3. What are your views on implementing a new CVD risk assessment and prevention activity in your PHN?
4. We would like to document any CVD risk assessment resources your PHN uses with General Practice. Does your PHN use:
5. Clinical audit & feedback reports on CVD risk assessment (prompt: Pen CS, POLAR)
6. HealthPathways section on CVD risk assessment
7. Local website with CVD risk assessment information
8. Local prevention/lifestyle programs involving CVD risk assessment (prompt: My Health For Life)
9. GP training (prompt: online modules or workshops for CPD points)
10. Other resources (prompt: apps, training)
11. Would it be possible to see de-identified examples of the above so we can compare how different PHNs promote CVD risk assessment?

**Part 3. Barriers and facilitators**

1. What CVD risk assessment tools are you aware of being used by GPs or practice nurses in the PHN? (prompt: existing tools eg cvdcheck.com.au, Best Practice/Medical Director, PIP QI app in Topbar)
2. What do you think are the main barriers to using these current tools? (prompt: capability, opportunity and motivation barriers eg knowledge, communication, access, time, attitudes)
3. What do you think might increase use of these tools? (prompt: planned activities in CVD prevention eg MBS items for Heart Health Check, PIP QI, new guidelines)
4. Are there any systemic barriers such as lack of reimbursement under Medicare or other programmes that prohibit your PHN from conducting CVD risk assessment or prevention activities?
5. Do you think that CVD risk assessment or prevention activities are well evaluated or audited in your PHN? (by how much, level of improvement? Ways to improve quality of data or number of practices engaged?)
6. Show resources at [www.auscvdrisk.com.au](http://www.auscvdrisk.com.au) to prompt any further ideas for implementation (topbar version).
