# Supplementary figures and images for: Implementing decision aids for cardiovascular disease prevention: stakeholder interviews and case studies in Australian primary care
Source: BMC Prim Care. 2024 Feb 3;25:49. doi: 10.1186/s12875-023-02258-4 (PMC10837956; doi:10.1186/s12875-023-02258-4)

***Additional figure 1 : Quality of audit and feedback reports***


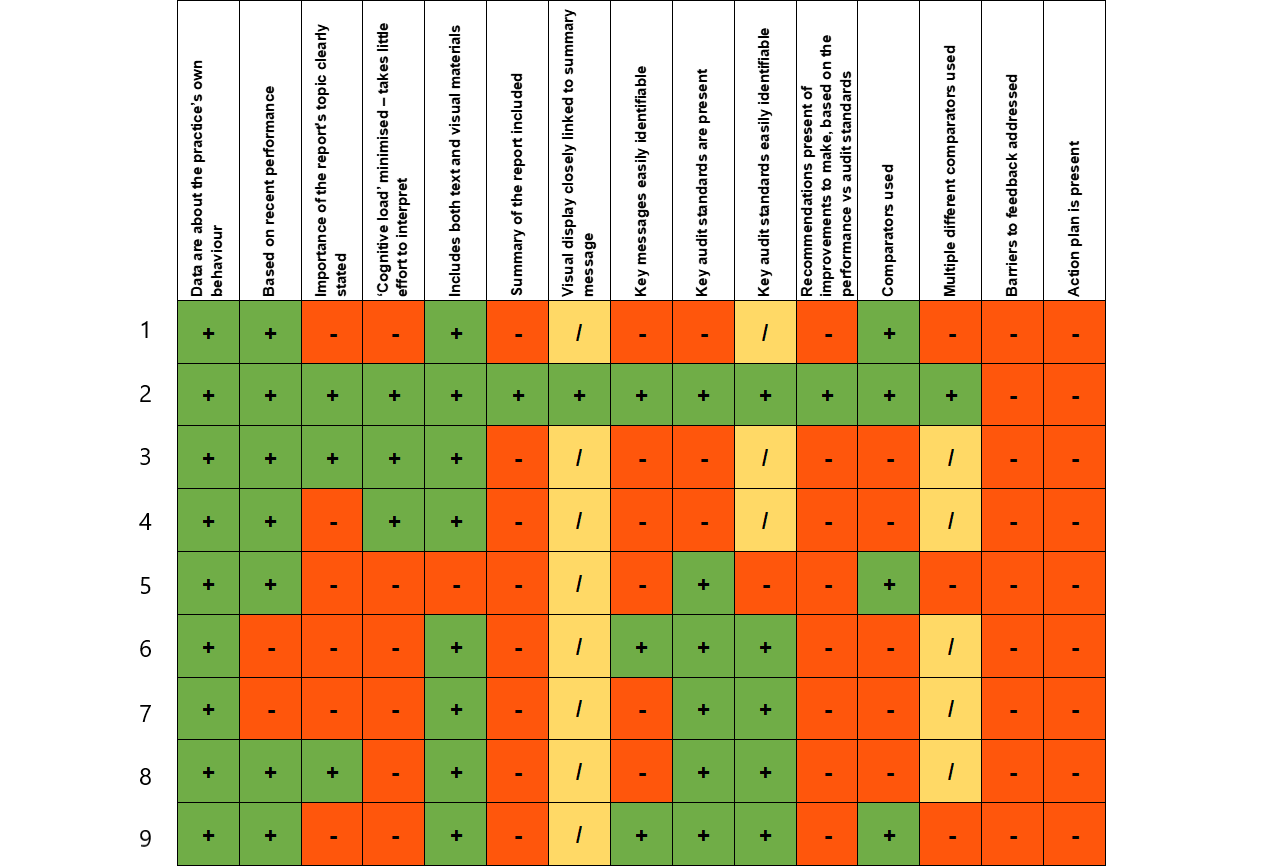


Green + = Yes, Red - = No, Yellow / = N/A

Supplement: Supplementary file 2 — Supplementary Material 2: Qualitative Checklist [file 12875_2023_2258_MOESM2_ESM.docx]
