# Supplementary material for: Implementing decision aids for cardiovascular disease prevention: stakeholder interviews and case studies in Australian primary care
Source: BMC Prim Care. 2024 Feb 3;25:49. doi: 10.1186/s12875-023-02258-4 (PMC10837956; doi:10.1186/s12875-023-02258-4)
Supplement: Supplementary file 3 — Supplementary Material 3: Staff Semi-structured Interview Guide [file 12875_2023_2258_MOESM3_ESM.docx]

***Table S1: Implementation study stages***

| **Stage** | **1: stakeholder consultation** | **2: stakeholder interviews** | **3: pilot testing implementation strategies** |
| --- | --- | --- | --- |
| Time period | 2018-2022 | 2020 | 2019-2021 |
| Participants | n=8 experts in general practice, behavioural science, cardiology biostatistics and health economics; including representatives of the Heart Foundation, RACGP and Primary Health Networks. | n=29 staff involved in managing or implementing general practice programs in PHNs in every state/territory (including 18 of the 31 PHNs in Australia). | Low resource (passive website link): the Heart Foundation & 1 PHN in New South Wales implemented links to the decision aid in clinician webpages. This was subsequently adopted by 16 other PHN webpages across 4 states.  Medium resource (active GP education): 1 PHN in Victoria ran a workshop on the guidelines & decision aid at a GP conference; 1 PHN in New South Wales provided the decision aid in audit and feedback reports for general practice QI.  High resource (GP software integration app): 9 PHNs across Queensland and New South Wales agreed to pilot an integrated app to auto-populate the decision aid from patient records with a single click. This was limited to a feasibility test in 4 practices due to COVID-19 disruptions. |
| Process | Monthly meetings in 2018-20 to map implementation opportunities and three hour workshop in 2022 to present pilot study findings from stage 3 and identify stakeholder priorities for implementing new CVD guidelines in 2023. | A snowballing recruitment method was used for online interviews with $50 gift voucher offered to participants. Interviews were audio recorded and transcribed. Staff from each of the 7 states/territories in Australia were recruited. | Opportunistic piloting of low, medium & high resource strategies in selected PHNs. Originally this was planned as a pilot adaptive trial starting with low resource strategies and intensifying over time; but the disruptions of COVID-19 to general practice required a more opportunistic case study approach as state-based outbreaks and restrictions were unpredictable at this time. |
| Data | Meeting discussion notes and actions circulated to participants after each meeting. | Audio recording transcripts analysed using Framework Analysis. | Descriptive app and Google analytics were used to evaluate user time trends to pilot test uptake and reach. |

*Note: CVD: cardiovascular disease; GP: general practitioner; PHN: Primary Health Network; QI: Quality Improvement; RACGP: The Royal Australian College of General Practitioners*
