# Supplementary material for: Implementing decision aids for cardiovascular disease prevention: stakeholder interviews and case studies in Australian primary care
Source: BMC Prim Care. 2024 Feb 3;25:49. doi: 10.1186/s12875-023-02258-4 (PMC10837956; doi:10.1186/s12875-023-02258-4)
Supplement: Supplementary file 4 — Supplementary Material 4: Additional figure 1 : Quality of audit and feedback reports [file 12875_2023_2258_MOESM4_ESM.docx]

| **Standard number** | **Description of the standard** | **Brief description of how standard is met or NA** | **Page and line number of where standard has been detailed** |
| --- | --- | --- | --- |
| 1 | Title | provided on first page | page 1 line 1 |
| 2 | Abstract | Covered in abstract | page 2 line 29 |
| 3 | Problem Description | Covered in background | page 4 line 74 |
| 4 | Available knowledge | Covered in background | page 4 line 74 |
| 5 | Rationale | Covered in background | page 4 line 74 |
| 6 | Specific aims | Covered in aim | page 6 line 164 |
| 7 | Context | Covered in context | page 7 line 215 |
| 8 | Intervention(s) | covered in methods | page 6 line 172 |
| 9 | Study of the Intervention(s) | covered in methods | page 6 line 172 |
| 10 | Measures | covered in methods | page 6 line 172 |
| 11 | Analysis | covered in methods | page 6 line 180 |
| 12 | Ethical considerations | covered in declarations | Page 23 line 562 |
| 13 | Results | covered in results | Page 9 line 293 |
| 14 | Summary | covered in summary | Page 17 line 415 |
| 16 | Interpretation | covered in discussion | pages 17-20 + tables |
| 19 | Limitation | covered in discussion | page 20 line 499 |
| 20 | Conclusions | covered in discussion | page 20 line 515 |
| 21 | Funding | covered in declarations | page 23 line 578 |

Supplementary file 1 : Checklist
