# Supplementary material for: Implementing decision aids for cardiovascular disease prevention: stakeholder interviews and case studies in Australian primary care
Source: BMC Prim Care. 2024 Feb 3;25:49. doi: 10.1186/s12875-023-02258-4 (PMC10837956; doi:10.1186/s12875-023-02258-4)
Supplement: Supplementary file 5 — Supplementary Material 5: Table S1: Implementation study stages [file 12875_2023_2258_MOESM5_ESM.docx]

| **Standard number** | **Description of the standard** | **Brief description of how standard is met or NA** | **Page and line number of where standard has been detailed** |
| --- | --- | --- | --- |
| **Title and abstract** | | | |
| 1 | Title | provided on first page | Page 1 line 1 |
| 2 | Abstract | provided on third page | Page 2 line 29 |
| **Introduction** | | | |
| 3 | Problem formulation | Covered in introduction | Page 4 line 73 |
| 4 | Purpose or research question | Covered in introduction | Page 6 line 164 |
| **Methods** | | | |
| 5 | Qualitative approach and research paradigm | Covered in methods | Page 6 line 175 |
| 6 | Researcher characteristics and reflexivity | Covered in methods | Page 7 line 208 |
| 7 | Context | Covered in setting | Page 7 line 215 |
| 8 | Sampling strategy | covered in methods | Page 6 line 175 |
| 9 | Ethical issues pertaining to human subjects | covered in ethics | Page 23 line 562 |
| 10 | Data collection methods | covered in methods | Page 6 line 175 |
| 11 | Data collection instruments and technologies | covered in methods | Page 6 line 181 |
| 12 | Units of study | covered in methods | Page 6 line 175 |
| 13 | Data processing | covered in methods | Page 6 line 180 |
| 14 | Data analysis | covered in methods | Page 6 line 180 |
| 15 | Techniques to enhance trustworthiness | covered in methods | Page 6 line 208 |
| **Results** | | | |
| 16 | Synthesis and interpretation | covered in results | Page 9 line 293 |
| 17 | Links to empirical data | quotes in results | Page 13 line 358 |
| **Discussion** | | | |
| 18 | Integration with prior work, implications, transferability, and contribution(s) to the field | covered in discussion | Page 17 line 413 |
| 19 | Limitations | covered in discussion | Page 20 line 499 |
| **Other** | | | |
| 20 | Conflicts of interest | covered in competing interests | Page 23 line 574 |
| 21 | Funding | covered in funding | Page 23 line 581 |

Supplementary file 2 : Qualitative Checklist
